# Small molecules enhance CRISPR/Cas9-mediated homology-directed genome editing in primary cells

**DOI:** 10.1038/s41598-017-09306-x

**Published:** 2017-08-21

**Authors:** Guoling Li, Xianwei Zhang, Cuili Zhong, Jianxin Mo, Rong Quan, Jie Yang, Dewu Liu, Zicong Li, Huaqiang Yang, Zhenfang Wu

**Affiliations:** 1 National Engineering Research Center for Breeding Swine Industry, College of Animal Science, South China Agricultural University, Guangzhou, 510642 China; 2 Guangdong Provincial Key Laboratory of Agro-animal Genomics and Molecular Breeding, College of Animal Science, South China Agricultural University, Guangzhou, 510642 China

## Abstract

CRISPR/Cas9 is an efficient customizable nuclease to generate double-strand breaks (DSBs) in the genome. This process results in knockout of the targeted gene or knock-in of a specific DNA fragment at the targeted locus in the genome of various species. However, efficiency of knock-in mediated by homology-directed repair (HDR) pathway is substantially lower compared with the efficiency of knockout mediated by the nonhomologous end-joining (NHEJ) pathway. Suppressing NHEJ pathway or enhancing HDR pathway has been proven to enhance the nuclease-mediated knock-in efficiency in cultured cells and model organisms. We here investigated the effect of small molecules, Scr7, L755507 and resveratrol, on promoting HDR efficiency in porcine fetal fibroblasts. Results from eGFP reporter assay showed that these small molecules could increase the HDR efficiency by 2–3-fold in porcine fetal fibroblasts. When transfecting with the homologous template DNA and CRISPR/Cas9 plasmid and treating with small molecules, the rate of knock-in porcine fetal fibroblast cell lines with large DNA fragment integration could reach more than 50% of the screened cell colonies, compared with 26.1% knock-in cell lines in the DMSO-treated group. The application of small molecules offers a beneficial approach to improve the frequency of precise genetic modifications in primary somatic cells.

## Introduction

The bacterial adaptive immune system clustered regularly interspaced short palindromic repeats (CRISPR)/CRISPR-associated protein 9 (Cas9) nuclease represents a versatile approach for genome engineering^1^. CRISPR/Cas9 system enables efficient and precise genetic alterations by inducing targeted DNA double-strand breaks (DSBs) that stimulate cellular DNA repair mechanisms, such as error-prone nonhomologous end-joining (NHEJ) and error-free homology-directed repair (HDR)^2^. NHEJ is characterized by introducing unpredictable patterns of insertions or deletions (indels) at the targeted site of genome DNA, thus enables the gene knockout of single or both alleles through the induction of frameshift mutations^3^. By contrast, HDR causes knock-in events that result in precise point mutations or insertion of a fragment of desired sequence at the targeted locus, such as codon replacements or reporter gene insertions, by recombination with exogenous homologous template^3^. In mammalian cells, NHEJ is more efficient than HDR. In proliferating human cells, NHEJ has been reported to repair 75% of DSBs, while HDR repaired the remaining 25%^4^, and the mouse embryonic stem cells showed a similar ratio^5^. The HDR efficiency following CRISPR/Cas9-induced DSB is also quite inefficient (0.5–20%) compared with the higher efficiency of NHEJ (which can reach up to 100%) in mammalian cells and mouse embryos^6–8^. Therefore, the low efficiency of CRISPR/Cas9-mediated precise gene editing remains a major challenge in generating cell lines or model organisms with desired mutation or correcting genetic mutation for gene therapy of human inherited disorders.

Previous study suggested a trade-off between the two DNA repair pathways after DSBs were created by Cas9 nuclease^9^. Inhibiting the expression or function of essential factors of NHEJ pathway would conversely improve the nuclease-mediated HDR efficiency. Various small molecules have been identified to enhance or repress NHEJ/HDR pathway to modulate the efficiency of CRISPR/Cas9-mediated genome alterations^9–11^. As an inhibitor of DNA ligase IV which is a key enzyme in the NHEJ pathway, Scr7 directly binds to the DNA binding domain of Ligase IV and thus interferes with the progression of NHEJ events^11, 12^. Scr7 increased the efficiency of HDR-mediated genome editing up to 19-fold using CRISPR/Cas9 in mammalian cells and mouse embryos^11^. However, the function of Scr7 in promoting HDR remains controversial. A study has demonstrated that Scr7 showed no significant impact on improving the HDR events in rabbit embryos^13^. Therefore, additional work is necessary to confirm the effects of Scr7 on increasing HDR efficiency. L755507, which was previously characterized as a β3-adrenergic receptor partial agonist^14^, could enhance CRISPR/Cas9-mediated HDR efficiency by 2–3-fold for large fragment integration in diverse mammalian cells and by approximately 9-fold for point mutations in human induced pluripotent stem cells^9^. Resveratrol, a small-molecule compound found in grapes, exhibits a wide range of biological activities, such as the protective effect in response to stress, injury, UV irradiation and fungal infection, as well as anti-tumor potential against various types of cancer^15, 16^. Resveratrol has been shown to down-regulate the expression of *LIG4*, *PRKDC*, *KU70*, and *KU80* in the NHEJ pathway (Che, J. unpublished master’s thesis, Soochow University, 2008). Therefore, resveratrol might promote HDR for its inhibitory effect on the NHEJ process. The effect of resveratrol on increasing HDR has not yet been reported.

As an important large animal model in agricultural and biomedical studies, pigs are genetically modified to present desirable traits of economic importance or mimic human diseases^17^. Precise introduction of a point mutation or a sequence fragment into the target locations of pig genome is usually needed to achieve this purpose. The porcine fetal fibroblast is a major donor cell type in animal cloning to generate genetically modified pigs. These cells have low transfection efficiency and age quickly when cultured *in vitro*, resulting in a time consuming and remarkably inefficient screening when knock-in fibroblasts are obtained. To address this issue, we investigated the effects and elucidated the mechanisms of three small molecules, namely, Scr7, L755507, and resveratrol, on CRISPR/Cas9-mediated HDR in porcine fetal fibroblasts.

## Results

### Screening of sgRNA with high cleavage efficiency to *ROSA26*

**Figure 1:**
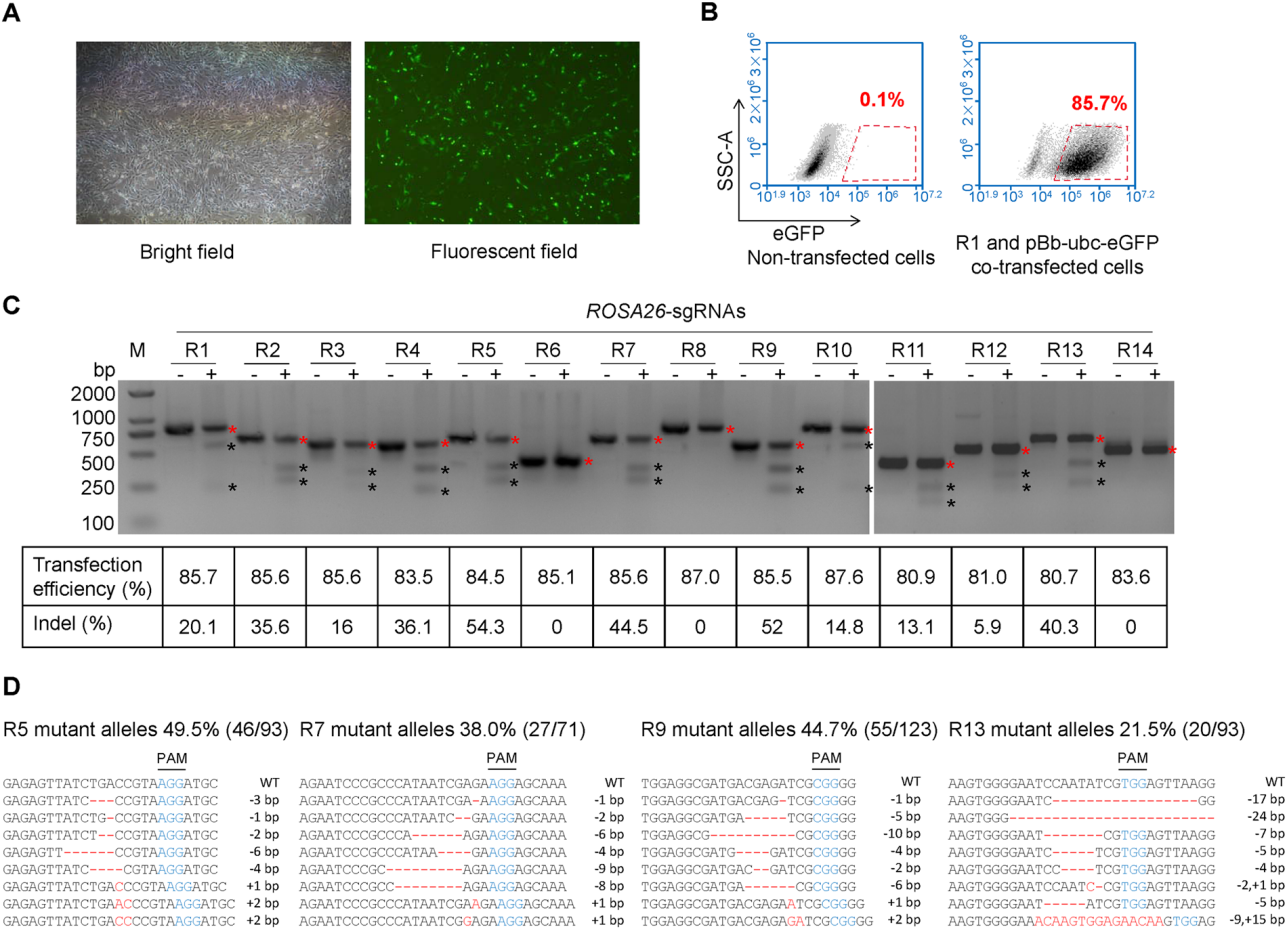
CRISPR/Cas9 induced targeted mutations at the *ROSA26* locus. (**A**) Fluorescence expression in porcine fetal fibroblasts co-transfected with sgRNAs and pBb-ubc-eGFP by electroporation. (**B**) Detection of transfection efficiency by flow cytometry at 48 h post-transfection. (**C**) T7E1 assay showing percentage of indel formations (+) in the 14 targeted sites of pig *ROSA26* locus. Control cells (−) were transfected with Cas9 only (without sgRNA). The transfection efficiency and indel percentages for each sgRNA are shown at the bottom. Representative images from n = 3 independent experiments. The red and black asterisks indicate uncut and cut bands by T7E1, respectively. (**D**) Sanger sequencing detected targeted mutation (indels) at R5, R7, R9, and R13 targeted sites in *ROSA26*. Eight mutant alleles are presented as representative of all the mutant alleles for each sgRNA transfected group. The fractions in the brackets indicate the ratios of mutant alleles to all sequenced alleles. Indels are shown in red letters and dashes. PAM sequence is shown in blue.

We designed 14 sgRNAs targeting *ROSA26* intron 1 region of the pig genome based on the N20NGG rule^7^. To evaluate the transfection efficiency of CRISPR-targeting plasmids (R1–R14) in porcine fetal fibroblasts, the CRISPR-targeting plasmids were co-transfected with a fluorescence expressing plasmid (pBb-ubc-eGFP), which has a similar size with the CRISPR plasmid, into the porcine fetal fibroblasts by electroporation. Flow cytometry showed that the percentage of fluorescence expressing fibroblasts was 85.7% in R1 and pBb-ubc-eGFP co-transfected fibroblasts (Fig. 1A and B), and all 14 sgRNAs had similar cotransfection efficiencies (Fig. 1C). After transfection with identical efficiency, T7EI assays demonstrated that some sgRNAs were highly efficient in cleaving the targeted loci. For example, R5, R7, R9, and R13 had cleavage rates of more than 40%. Several sgRNAs showed lower efficiency or no cleavage activity (Fig. 1C). To further confirm the cleavage activities of the sgRNAs, the targeted sites of R5, R7, R9, and R13 were amplified by PCR and then subjected to TA cloning and Sanger sequencing. The rates of mutant alleles harboring indels at cleavage sites were 49.5%, 38.0%, 44.7%, and 21.5% for the cells transfected with R5, R7, R9, and R13, respectively (Fig. 1D). R5 sgRNA had the highest cleavage rate among the 14 tested sgRNAs. Thus, the CRISPR plasmid expressing R5 sgRNA (pX330-R5) was used in the subsequent experiments.

### Effects of small molecules on cell viability and cell cycle

**Figure 2:**
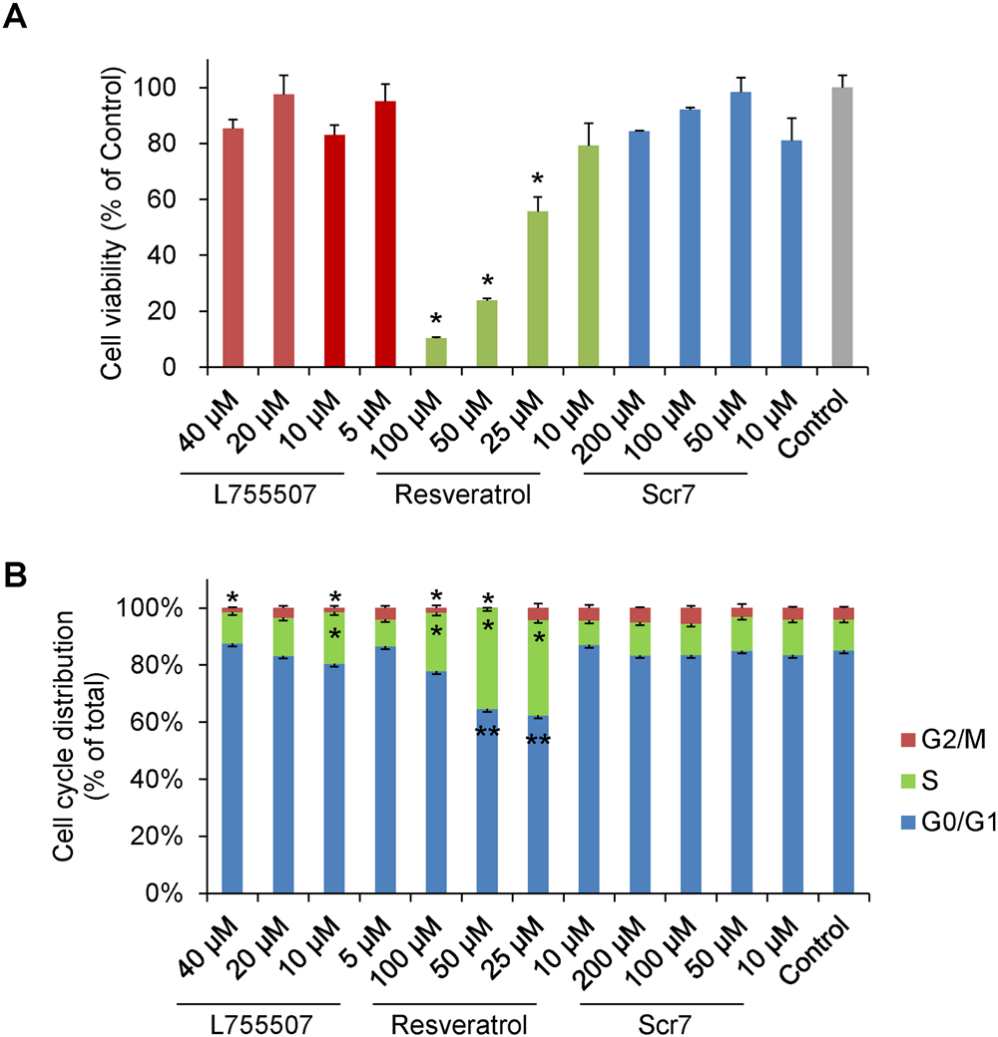
Effects of small molecules on cell viability and cell cycle. Effects of small molecules (Scr7, L755507, and resveratrol) on (**A**) cell viability and (**B**) cell cycle. DMSO-treated cells were used as control. Values are shown as mean ± SEM. An asterisk represents *P* < 0.01 and double asterisks represent *P* < 0.05 versus DMSO-treated control.

We investigated the effects of three small molecules, Scr7, L755507, and resveratrol, on CRISPR/Cas9-induced HDR. First, the effects of the three small molecules on cell viability and cell cycle were measured. Porcine fetal fibroblasts were equally distributed and grown in a 96-well plate and treated with small-molecule compounds at the indicated concentrations for 48 h. Control cells were treated with DMSO for the same time periods. Cell viability were determined using the MTT assay and cell cycle distribution were analyzed by flow cytometry after 48 h treatment. L755507 and Scr7 did not reduce cell viability significantly (Fig. 2A). By contrast, Resveratrol demonstrated severe cellular toxicity and significantly reduced cell viability. The reducing effect increased with increasing concentration of resveratrol (Fig. 2A). Scr7 did not affect cell cycle distribution in a range of 10–200 μM. L755507 significantly decreased the proportion of cells in the G2/M phase at 10 μM or 40 μM and increased the S-phase cells at 10 μM compared with the DMSO-treated cells. Upon treatment with 25–100 µmol/l resveratrol, the S-phase cells were increased significantly. S phase accumulation was accompanied by concomitant reduction in G0/G1-phase cells and G2/M-phase cells (Fig. 2B).

### Expression level of NHEJ and HDR key factors after treatment with small molecules

**Figure 3:**
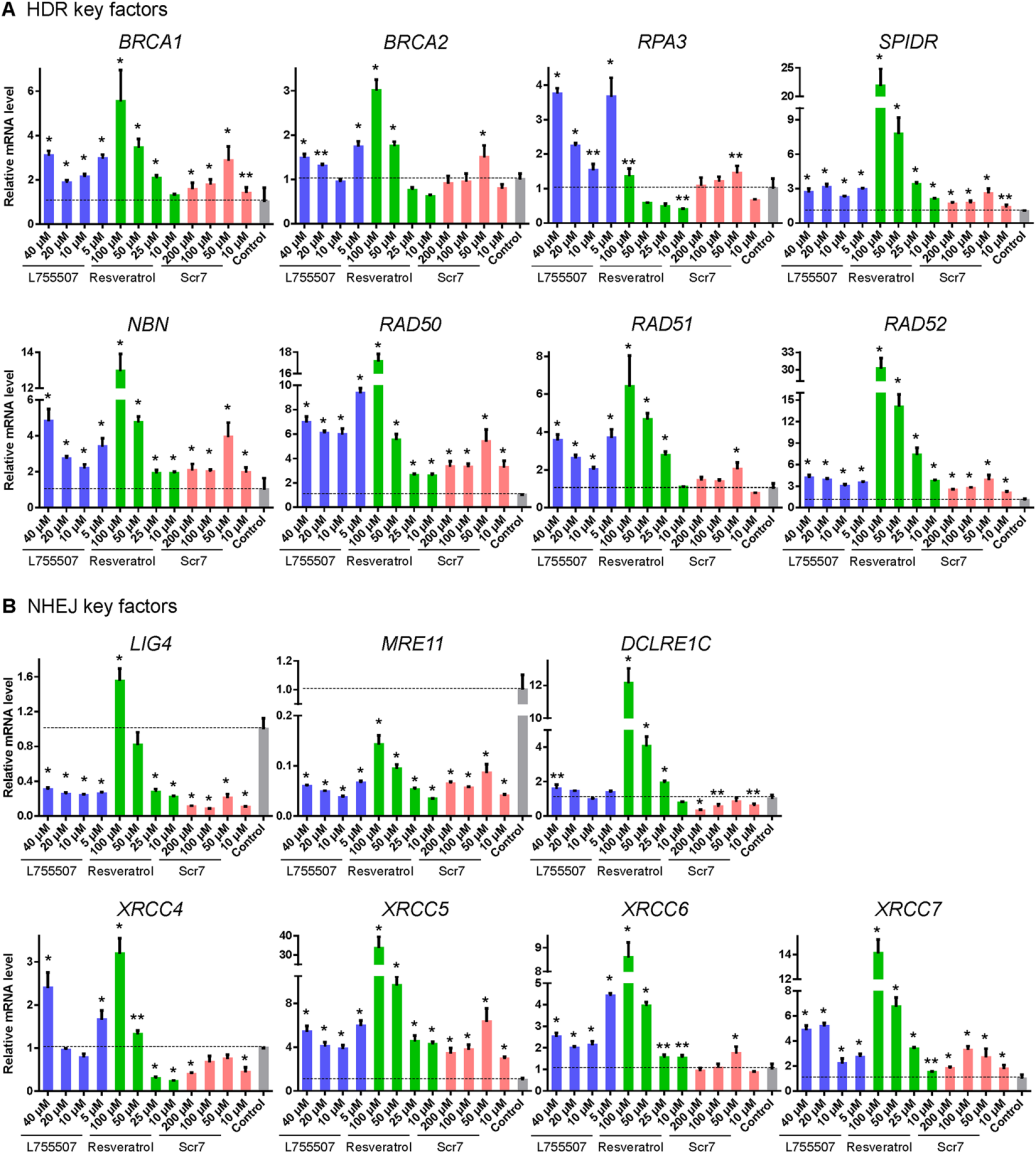
Effects of small molecules on the gene expression in homology-directed repair (HDR) and nonhomologous end-joining (NHEJ) pathways. Effects of small molecules on the gene expression of (**A**) HDR and (**B**) NHEJ key factors. DMSO-treated cells were used as control. Values are shown as mean ± SEM. An asterisk represents *P* < 0.01 and double asterisks represent *P* < 0.05 comparing the control.

We next detected the mRNA levels of NHEJ and HDR key factors in the porcine fetal fibroblasts after treatment with small molecules at the indicated concentrations for 48 h to examine the influence of small molecules on the expression of key factors in NHEJ and HDR pathways. *LIG4*, *MRE11*, *DCLRE1C*, *XRCC4*, *XRCC5*, *XRCC6*, and *XRCC7* in NHEJ pathway and *BRCA1*, *BRCA2*, *RPA3*, *SPIDR*, *NBN*, *RAD50*, *RAD51*, *RAD52* in HDR pathways were chosen for their crucial roles in regulating DNA repair pathways. Almost all key factors of HDR pathway in the cells treated with small molecules showed upregulated expression compared with the DMSO-treated control cells (Fig. 3A). Some NHEJ key factors, such as *LIG4* and *MRE11*, demonstrated significant reduction in mRNA expression level after treatment with L755507 or Scr7, but *XRCC5*, *XRCC6* and *XRCC7* showed upregulated expression in the cells treated with all the three compounds. It is interesting that resveratrol had dosage-dependent effects on the expression levels of some NHEJ factors. Low concentration (less than 25 μM) of resveratrol could inhibit the expression of *LIG4* and *XRCC4*. However, 50 μM resveratrol had no effect on the expression of *LIG4* or slightly up-regulated the expression of *XRCC4* and 100 μM resveratrol significantly promoted their expression (Fig. 3B).

### Small molecules increase the HDR efficiency and knock-in events

**Figure 4:**
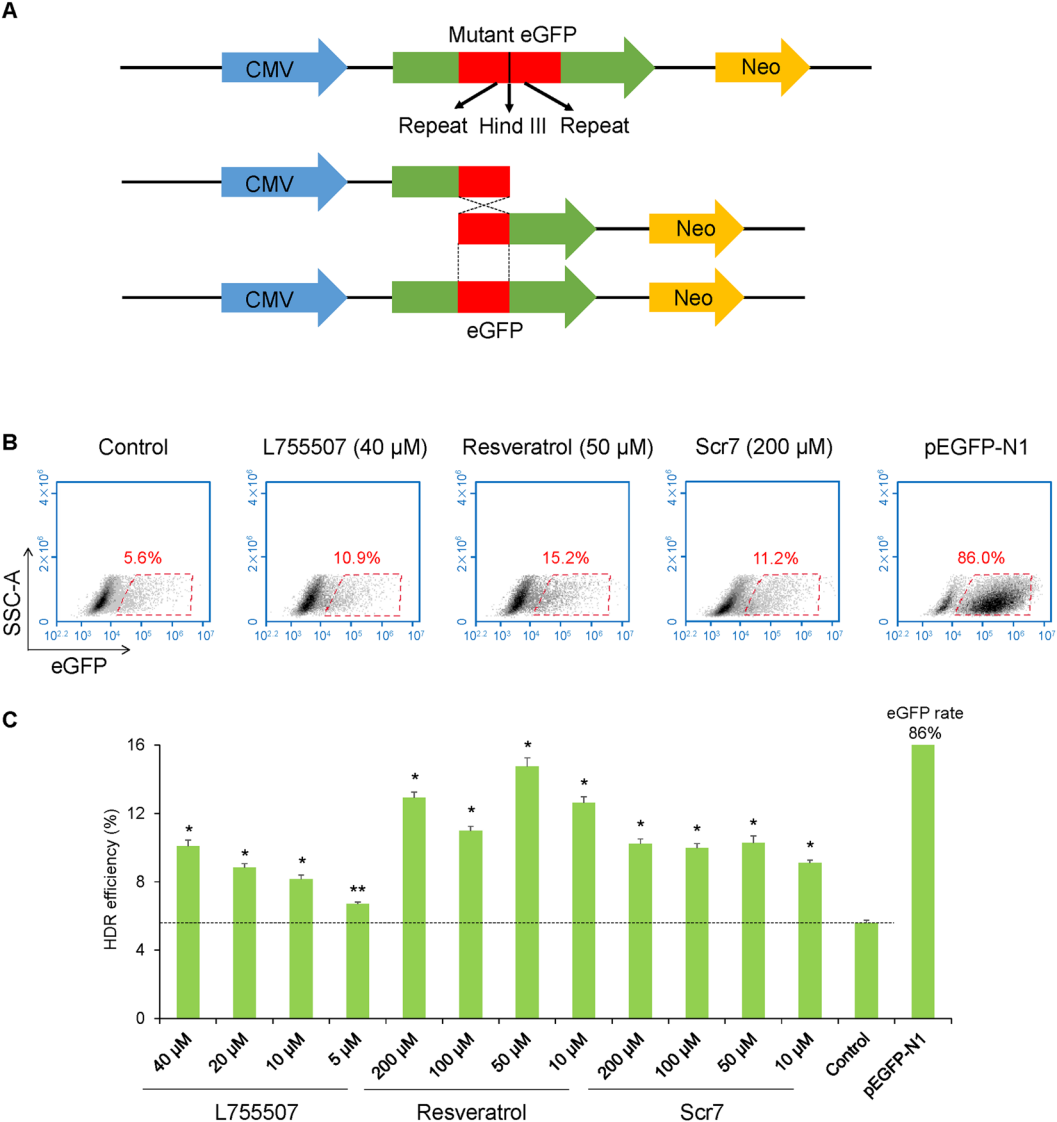
Effects of small molecules on HDR efficiency. (**A**) Schematic diagram of the eGFP reporter plasmid. (**B**) Detection of HDR efficiency in porcine fetal fibroblasts transfected the eGFP reporter by flow cytometry. (**C**) Summary of HDR efficiency in porcine fetal fibroblasts treated with different concentrations of the three small molecules. DMSO-treated reporter transfected cells were used as control. pEGFP-N1 transfected cells were used to set a gate for analyzing eGFP-positive cells. Values are shown as mean ± SEM. An asterisk represents *P* < 0.01, and double asterisks represent *P* < 0.05 versus control.

We constructed an eGFP reporter plasmid to evaluate the HDR efficiency in porcine fetal fibroblasts. The reporter system was established based on the HDR-mediated restoration of the mutated eGFP gene. The reporter plasmid contained two 200-bp repeats in the same direction flanking the HindIII restriction site. Upon nuclease cleavage, the generated DSB could be repaired by single strand annealing (SSA), a form of HDR, directed by the overlapping repeated sequences. The SSA process between the repeated sequences reconstituted a functional eGFP coding sequence (Fig. 4A). After digestion by HindIII, electroporation, and treatment with small molecules, flow cytometry assays showed that 40 μM L755507 and 50 μM resveratrol had the maximal effect on inducing HDR-mediated eGFP expression, and 50–200 μM Scr7 showed almost the same optimal activity on promoting HDR efficiency. Up to 10.9%, 15.2%, and 11.2% of transfected cells were positive for eGFP expression in L755507, resveratrol, and Scr7 treatment groups, respectively. The DMSO treatment control group only contained 5.6% eGFP-positive cells (Fig. 4B). On average, The HDR efficiency in the presence of 40 μM L755507 or 50–200 μM Scr7 was improved by approximately 2-fold compared with the untreated control. In the presence of 50 μM resveratrol, the HDR efficiency was improved by approximately 3-fold (Fig. 4C).

**Figure 5:**
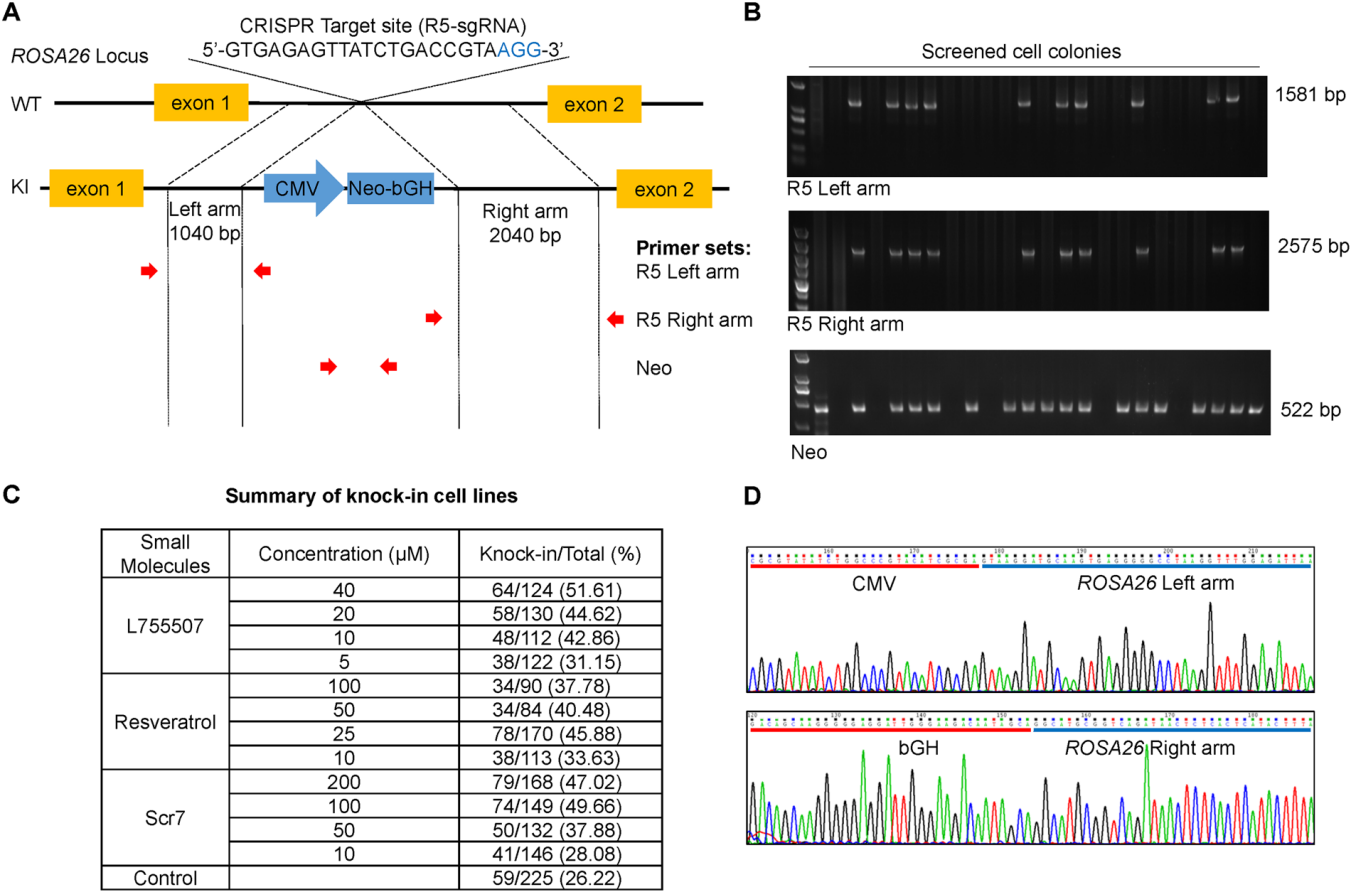
Effects of small molecules on the screening of knock-in porcine fetal fibroblasts cell lines. (**A**) Schematic diagram of the *ROSA26* HDR template vector and strategy for insertion of the neomycin resistance gene into the pig *ROSA26* intron 1 region. Red arrows are the binding sites of the primer sets to detect the insertion of the neomycin resistance gene to the targeted site. (**B**) Detection of knock-in cell lines using the primer sets shown in **A**. (**C**) Improvement of the percentage of knock-in cell lines in the presence of the three molecules in porcine fetal fibroblasts. Total represents the number of picked-up cell lines in each group, and Knock-in represents the number of knock-in cell lines identified by PCR and sequencing. (**D**) Representative sequencing results of junction regions of the inserted fragment at the targeted site in knock-in cell lines. Sequencing confirms precise insertion of neomycin resistance gene to targeted site of *ROSA26* intron 1 region.

We designed a donor vector to insert a Neomycin resistance gene (Neo) into the locus of *ROSA26* to further test how the small molecules can be used to generate knock-in primary porcine cell lines. The donor vector contained a left homology arm of 1040 bp and a right homology arm of 2040 bp flanking the CMV-Neo expressing cassette (1997 bp) (Fig. 5A). Porcine fetal fibroblasts were co-transfected with the donor vector and pX330-R5 and screened using 400 μg/ml G418 for two weeks. Single cell colonies were picked up for further culture and genotyping. The primer sets which were respectively located at genome and donor vector regions adjacent to the homology arm ends were used to amplify the homology arms for identifying the knock-in cell lines (Fig. 5A). Following PCR amplification, agarose gel electrophoresis for knock-in events showed that L755507, resveratrol, and Scr7 treatments could generate up to 51.61%, 45.88%, and 49.66% CRISPR/Cas9-mediated knock-in cell lines, respectively, in all picked-up cell lines, whereas the control cells treated with DMSO only generated 26.22% CRISPR/Cas9-mediated knock-in cell lines (Fig. 5B and C). DNA sequencing confirmed the CMV-Neo cassette was precisely inserted into the target site in the knock-in cell lines (Fig. 5D).

## Discussion

Recent developments in CRISPR/Cas9-mediated genome editing enable the efficient introduction of DSBs into specific locations in genomes. Given the cytotoxic lesions that threaten genomic integrity, DSBs can be repaired by employing two main mechanisms, NHEJ and HDR, which cause knockout or knock-in event in the targeted loci of genome, respectively^3, 18^. CRISPR/Cas9 system could achieve more than 60% biallelic knockout rate in the primary porcine fetal fibroblasts and 78% biallelic modification efficiency in mouse embryos^19, 20^. By contrast, the knock-in event, resulting in precise genome editing, is substantially low with the rate ranging from 0.5% to 20%^6, 7^. The cell will choose one from these pathways to respond to DSBs. The choice strategy was apparently not randomly performed. NHEJ repair pathway is often selected to generate a genetic knockout when utilizing CRISPR/Cas9. Thus, the comprehensive mechanisms regulating repair pathway choice should be further elucidated.

The two repair pathways differ in their demands for the homologous sequence and in the reliability of DSB repair. NHEJ is the predominant DSB repair pathway in nearly all species operating in all four cell cycle phases, whereas HDR is confined to the late S and G2 phases when DNA replication is completed and sister chromatids are readily available as repair templates^3, 18^. NHEJ entails elimination of DSB by direct ligation of DNA termini, therefore usually causes error-prone DNA repair. The initial step in NHEJ process involves recognition and binding of the KU70-KU80 heterodimer (KU) to the broken DNA ends of DSB. The Ku-DNA complex then recruits the catalytic subunit of the DNA-dependent protein kinase (DNA-PKcs) to generate the DNA-PK holoenzyme (KU-DNA-PKcs complex). The two DNA end-bound holoenzymes synapse, phosphorylate each other and dissociate from the DNA ends, facilitating the accessibility of the broken DNA ends. In addition, DNA-PKcs phosphorylates Artemis, which then acquires endonuclease activity that excises single-stranded overhangs of the broken ends. Finally, the complex of Ligase IV, XRCC4, and XLF tethers the two DNA ends. Compared with the NHEJ process, the typical HDR pathway, homologous recombination (HR), is more complex, requiring a homologous template DNA and resulting in the seamless correction of DSB. During the HR process, the DSB repair proteins MRE11, RAD50, and NBS1 form MRE11-RAD50-NBS1 complex and recognize and trim DSB to create 3′ overhangs of single-strand DNA. After end resection, RPA binds to single-stranded DNA ends to eliminate disruptive secondary structures, and is subsequently substituted by RAD51 in conjunction with several mediator proteins. The RAD51-coated single-stranded DNA tail then searches for homologous chromosomes or DNA chain to achieve homologous recombination^3, 18^. The alternative HDR pathway, SSA is initiated by an identical mechanism as HR and goes a separate way only after the DNA end resection. SSA results in removal of one of the annealed homologous sequences and thus is a non-conservative method to repair DSB. SSA probably serves as a backup for HR^3^.

These repair pathways apparently exhibit complementary roles. The efficiency of HDR could be promoted by inhibiting NHEJ key molecules or stimulating HDR key molecules^9, 11, 13^. Suppression of KU70 and DNA ligase IV promoted the efficiency of HDR by 4–5-fold in mammalian cells^21^. The small-molecule enhancer of HDR, RS-1, increased the knock-in efficiency by 2–5-fold at different loci in animal embryos^13^. Similar effects were also observed in Drosophila embryos^22^. The frequency of HDR declined significantly in flies with homozygous mutations in *spnA* (Drosophila ortholog of *RAD51*) or *okr* (Drosophila ortholog of *RAD54*), which are key molecules in HDR pathway, whereas the HDR frequency rose significantly in flies with genetic deficiency in *Lig4* ^22^.

In the three small-molecule compounds used in the present study, Scr7 and L755507 have been previously reported to enhance the HDR rate in diverse mammalian cells^9, 11, 21^. The effect of resveratrol on the HDR efficiency has not been reported yet. Scr7 and L755507 did not impair cell viability of porcine fetal fibroblasts, whereas resveratrol demonstrated significant cytotoxic action in a dosage-dependent manner. Additionally, L755507 and resveratrol could arrest cells at the S phase. As these small molecules could promote HDR, the expression levels of key genes in HDR pathway might be upregulated after small-molecule treatment. The Q-PCR results conformed to previous expectations. The mRNA levels of key factors of HDR pathway, namely, *BRCA1*, *BRCA2*, *RPA3*, *SPIDR*, *NBN*, *RAD50*, *RAD51*, and *RAD52* were increased significantly in cells treated with all the three compounds. The down-regulated mRNA expression of NHEJ factors was also expected. The Q-PCR results showed significant down-regulation of many NHEJ factors, such as *LIG4*, *MRE11*, *DCLRE1C*, and *XRCC4* in Scr7-treated fibroblasts, *LIG4* and *MRE11* in L755507-treated fibroblasts. The selective regulation of various NHEJ factors implies that these small molecules may employ different mechanisms to regulate the DNA repair pathways. Meanwhile, Some NHEJ factors in the presence of small-molecule compounds showed significant upregulation, such as *XRCC5*, *XRCC6*, and *XRCC7*. High concentration of resveratrol also caused significant upregulation of most NHEJ factors. A detailed mechanism for DNA repair pathways regulation by small molecules needs to be investigated further.

Two strategies were employed to measure the CRISPR/Cas9-mediated HDR efficiency in porcine fetal fibroblasts. One strategy is an eGFP reporter system which enables a single-strand annealing type of HDR to restore the eGFP expression in HDR-occurring and -occurred cells. Results from the eGFP reporter system demonstrated L755507 and Scr7 led to a 2-fold increase in HDR efficiency, while resveratrol led to a 3-fold increase in HDR efficiency in porcine fetal fibroblasts. Another strategy is the screening of knock-in fibroblasts using these small molecules to explore the general use of these compounds to establish genetically modified cell lines. After treatment with small molecules, approximately twice as much knock-in cell lines as that of DMSO-treated cells were obtained. These small molecules could significantly improve the efficiency of integrating large DNA fragment into the target site of pig genome to generate cell lines with seamless genetic modification.

Therefore, Scr7, L755507, or resveratrol could improve the knock-in efficiency by multifold in porcine fetal fibroblasts. This process remarkably reduced the workload in generating genetically modified cell line for somatic cell nuclear transfer to establish pig models with precisely modified genome. Furthermore, these small-molecule compounds could be used in combination with other strategies, such as synchronization of cell cycles^23^ or optimization of donor DNA design^24^, to further enhance the HDR efficiency. This process facilitates the seamless genetic modification on both alleles and even multiple genes.

## Materials and Methods

### Plasmid construction

The Cas9 and sgRNA co-expression plasmid pX330 was obtained from Addgene (Plasmid #42230). The sgRNAs were designed using E-CRISP tool (http://www.e-crisp.org/E-CRISP/) (Table 1). The sgRNAs were synthesized as DNA oligonucleotides and cloned into pX330 to form the CRISPR targeting plasmids pX330-R1–pX330-R14 (or R1–R14 for short). The eGFP reporter plasmid was constructed by dividing the eGFP coding sequence into two segments, which were separated by a stop codon and a HindIII restriction site. Both eGFP segments contained a 200 bp homology region (Fig. 4A). When the two repeat sequences recombine to restore eGFP function, the cells will resume fluorescence expression. HDR donor vectors were constructed by In-Fusion HD Cloning Kit (Takara, Dalian, China). The *ROSA26* homologous template in the donor consisted of the 1040 bp left arm and the 2040 bp right arm amplified from pig genome, flanking a 1997 bp CMV-Neo-bGH expression cassette (Fig. 5A).

**Table 1:** The sequences of sgRNAs^7, 25^.

Name	Strand	Sequences (5′–3′)
sgRNA-1 (R1)	F	CACCAGGTGGTCTGACCGGTAGCG
	R	AAACCGCTACCGGTCAGACCACCT
sgRNA-2 (R2)	F	CACCGAGCATATCGTTTGTTACGC
	R	AAACGCGTAACAAACGATATGCTC
sgRNA-3 (R3)	F	CACCAATGGCTCCGTCCGTATTCC
	R	AAACGGAATACGGACGGAGCCATT
sgRNA4 (R4)	F	CACCAACCACTACGCGAGTCGGCA
	R	AAACTGCCGACTCGCGTAGTGGTT
sgRNA-5 (R5)	F	CACCGTGAGAGTTATCTGACCGTA
	R	AAACTACGGTCAGATAACTCTCAC
sgRNA-6 (R6)	F	CACCAGGCGATGACGAGATCGCGG
	R	AAACCCGCGATCTCGTCATCGCCT
sgRNA-7 (R7)	F	CACCAATCCCGCCCATAATCGAGA
	R	AAACTCTCGATTATGGGCGGGATT
sgRNA-8 (R9)	F	CACCAGATTGAACCTACAACCTCG
	R	AAACCGAGGTTGTAGGTTCAATCT
sgRNA-9 (R8)	F	CACCTGGAGGCGATGACGAGATCG
	R	AAACCGATCTCGTCATCGCCTCCA
sgRNA-10 (R10)	F	CACCGGAGGCGATGACGAGATCGC
	R	AAACGCGATCTCGTCATCGCCTCC
sgRNA-11 (R11)	F	CACCGGCAAGTCTGCCATACTAAC
	R	AAACGTTAGTATGGCAGACTTGCC
sgRNA-12 (R12)	F	CACCCTAAGTAAGGGATACCGACT
	R	AAACAGTCGGTATCCCTTACTTAG
sgRNA-13 (R13)	F	CACCAAGTGGGGAATCCAATATCG
	R	AAACCGATATTGGATTCCCCACTT
sgRNA-14 (R14)	F	CACCGAATCCGACTAGGTACCGTG
	R	AAACCACGGTACCTAGTCGGATTC

### Cell culture and electroporation

Porcine fetal fibroblasts were maintained in DMEM (Life Technologies) supplemented with 15% fetal bovine serum (Life Technologies). The fibroblasts were electroporated with the Nucleofector 2b Device with an Amaxa kit following the manufacturer’s instructions (Lonza, Basel, Switzerland). In brief, cells were harvested with 0.25% trypsin (Life Technologies), centrifuged at 300 × *g* for 5 min at room temperature. We then carefully resuspended the cell pellet [(0.5–1) × 10^6^ cells per sample] in 100 μL of nucleofector solution (containing 4 μg of eGFP reporter plasmid or 4 μg of pEGFP-N1 for HDR rate evaluation in eGFP reporter assay, and 8 μg of CRISPR/Cas9 plasmid and 8 μg of HDR donor for knock-in cell lines screen). Then, the cell/plasmid mixture was transferred into a cuvette and program A-033 was used for nucleofection. Following transfection, 500 μL of the pre-equilibrated culture media was added to the cuvette, and the sample was immediately transferred gently into the 6-well plate.

### PCR amplification of target region

The intron 1 of *ROSA26* locus, containing 14 target sites, was PCR-amplified using the primer sets listed in Table 2. PCR reaction was performed using 200 ng of genomic DNA template and PrimeSTAR Max DNA polymerase (Takara, Dalian, China) according to the manufacturer’s protocol. The thermocycler setting consisted of one cycle of 98 °C for 5 min, 30 cycles of 98 °C for 10 s, 62 °C for 5 s, and 72 °C for 10 s, and one cycle of 72 °C for 5 min. The PCR products were analyzed on 2% agarose gel.

**Table 2:** Primer information.

Name	Strand	Sequences (5′–3′)	Product size (bp)
*ROSA26*-sgRNA-1	F	CAGGGGCACCCGGGACAC	816
	R	CTCCCTGCCGACTCGCGTA	
*ROSA26*-sgRNA-2	F	CAGGGGCACCCGGGACAC	659
	R	GGAGCCACTTTCACTGACCCTC	
*ROSA26*-sgRNA-3	F	TGGGGCTCCGGCTCCTC	604
	R	ATCAGATACCAAAGCCGAGCA	
*ROSA26*-sgRNA-4	F	CTTCTCCTCCCGCCGTGTG	610
	R	CAGGACCGACCCCCCACTCA	
*ROSA26*-sgRNA-5	F	AGATCTTTGTGTCGCAATTTCC	633
	R	CCAGCAACACCTAAGATTTATCAGA	
*ROSA26*-sgRNA-6	F	GAGTGCCGCAATACCTTTATGGGA	407
	R	AAACTCAGTAGATCCGTGCTT	
*ROSA26*-sgRNA-7	F	CTACCTGCTCTCGGACCCGTG	665
	R	GGCCTAAGGTTTGGAGATT	
*ROSA26*-sgRNA-8	F	TTGGTGGCCTAGAAATCACA	823
	R	AATAAAGCCTGAGGAAGTATAGCAC	
*ROSA26*-sgRNA-9	F	GCGCAACGTGGCAGGAAG	615
	R	AATGCATAAAATCAGGCTTAGGTG	
*ROSA26*-sgRNA-10	F	CTACCTGCTCTCGGACCCGTG	843
	R	TCAAGATGGGTAGTGTTAATTGG	
*ROSA26*-sgRNA-11	F	TCCTGCTGACAACAGTAACAC	433
	R	CCCTAAGCTAAGTAAGGGATA	
*ROSA26*-sgRNA-12	F	ATAGAATTCGTTATTTTACTAGC	603
	R	TTCTTTTCAGTGACTGGTGT	
*ROSA26*-sgRNA-13	F	CTTAGCTTAGGGTACCATGT	694
	R	CCACAGAATGTATTTAACTACAGAG	
*ROSA26*-sgRNA-14	F	TTGGGCTTGTTATGTTGCT	529
	R	TGTACTCACGGTCCAGATTTA	
*LIG4*	F	GCCGCTATCGCAGACATTG	251
	R	GCCATCATCTCACCATCAAGG	
*XRCC4*	F	AGCATTGGTGTCAGGAGCAG	184
	R	GGTGTCCAGGCAGTAACAAATAAG	
*XRCC5*	F	GAGGAAGGCACCGTTGAAG	188
	R	GAGAGAGGAATCTGACACTTAGC	
*XRCC6*	F	GCGATGAAGAAGAAGAAGAGGAG	225
	R	CATAGAACACCACTGCCAAGAG	
*DCLRE1C*	F	GTCTCCTTATACTGTTCACCTGTTAC	146
	R	TCTTCCTTCTCGCCTGATGC	
*XRCC7*	F	GCAGAATTATTCCAGCATTGATG	129
	R	TGGGACGATAAATTACCTTGTTTG	
*MRE11*	F	ATGACTTCCTTGACCTTGTTATCTG	146
	R	ATGTTTCTTTACCGCTTCTCCTG	
*RAD50*	F	GTGGTGATGCTAAAGGGAGAC	232
	R	GGAAGTTACGCTGCTGTGAG	
*NBN*	F	TCAGTCTAAGAAACACCCTCCAG	195
	R	GCATCTCCTCCTCCAAATACAAC	
*RPA3*	F	TCGTAGGGAGGCTGGAGAAG	167
	R	GACACACATAATGGTTGCTTTGC	
*RAD51*	F	CGTTCAACACAGACCACCAG	187
	R	GCAAGTCGCAGAAGCATCC	
*RAD52*	F	CTACTGGTGGCAACTCTGTATTATG	163
	R	ACCCTGTGACCCTCAATGTAAC	
*BRCA1*	F	ACGCCACTCTCAACTTCTG	195
	R	CAAGCCTGATGCCACAATAG	
*BRCA2*	F	GAGGAGGAGGAGGAGGATG	190
	R	ATCTGTTAGTTCTGCTGTGTTC	
*SPIDR*	F	CGAGGAAGGATGGCAGAAC	143
	R	GCAGTGAGGATATAACAGAAGC	
*ACTB*	F	CCACGTTACTACCTTCTTCTC	131
	R	TGATCTCCTTCTGCATCCTGT	
Neo	F	GCGTGGATAGCGGTTTGAC	522
	R	GCCGATTGTCTGTTGTGCC	
R5 Left arm	F	ATGTTCCCATAGTAACGCCAATA	1581
	R	GGTGCAGATGAACTTCAGGGT	
R5 Right arm	F	CAGCCATCTGTTGTTTGCC	2575
	R	ACTTGGAGTTCCCATCGTG	

### T7 endonuclease I assay

T7 endonuclease I recognizes and cleaves mismatched heteroduplex DNA which arises from the hybridization of wild-type and mutant DNA strands. The hybridization reaction contained 200 ng of PCR DNA in NEBuffer 2 and was performed on a thermocycler with the following settings: 95 °C, 10 min; 95–85 °C at −2 °C/s, 85 °C for 1 min, 85–75 °C at −0.1 °C/s, 75 °C for 1 min, 75–65 °C at −0.1 °C/s, 65 °C for 1 min, 65–55 °C at −0.1 °C/s, 55 °C for 1 min, 55–45 °C at −0.1 °C/s, 45 °C for 1 min, 45–35 °C at −0.1 °C/s, 35 °C for 1 min, 35–25 °C at −0.1 °Cs, 25 °C for 1 min, and held at 4 °C. Five units of T7 endonuclease I (NEB, Ipswich, MA, USA) were added to digest the re-annealed DNA at 37 °C for 1 h. The DNA fragments were separated on 2% agarose gel. The DNA band intensity was quantitated using Image Lab (Bio-rad, Hercules, CA, USA). Cleavage activity was calculated as {1−[1−(*b* + *c*/*a* + *b* + *c*)]1/2} × 100, where *a* is the band intensity of DNA substrate, and b and c are the cleavage products.

### Small-molecule compounds treatment

Approximately 1 × 10^4^ or 1 × 10^6^ porcine fetal fibroblasts were seeded on 96-well plates or 6-well plates for assays. After overnight incubation, cells were treated with small molecules at the indicated concentrations for 48 h before analysis. L755507 (Amquar, Shanghai, China) was used at 5 μM, 10 μM, 20 μM, and 40 μM and Scr7 (Amquar, Shanghai, China) was 10 μM, 50 μM, 100 μM, and 200 μM for all assays. Resveratrol (Sigma, St. Louis, MO, USA) was 10 μM, 25 μM, 50 μM, and 100 μM for analyses of cell viability, cell cycle distribution, mRNA expression, and knock-in cells selection; 10 μM, 50 μM, 100 μM, and 200 μM for eGFP reporter-based HDR efficiency assay.

### MTT assay

Cell viability was determined colorimetrically using the 3-(4,5-dimethylthiazol-2-yl)-2,5-diphenyltetrazolium bromide (MTT) reagent (Sigma, St. Louis, MO, USA). The cells were evenly added to a 96-well plate and incubated at 37 °C. After 48 h treatment with small molecules at the indicated concentrations, we carefully washed the cells with PBS 2–3 times and added MTT solution (5 mg/mL) to each well. The plates were incubated at 37 °C for 2.5 h. The supernatant was discarded, and the formed formazan crystals were dissolved in isopropanol. The color intensity was determined at 560 nm with a Synergy HT Multi-Mode Microplate reader (BioTek, Winooski, VT, USA).

### Analysis of cell cycle distribution

After 48 h of treatment with small molecules, fibroblasts were harvested, resuspended in PBS, and then fixed in cooled 70% ethanol at 4 °C for 30 min. The cells were incubated in a solution containing 1 mg/mL RNase A, 20 μg/mL propidium iodide (Sigma), and 0.05% Triton X-100 (Sigma) at 37 °C for 40 min. Subsequently, the cells were pelleted and resuspended again in PBS. Cell cycle was analyzed in the Accuri C6 Flow Cytometer (BD Biosciences, San Jose, CA, USA). At least 10,000 cells per sample were counted per run, and the proportion of cells in G0/G1, S, and G2/M phases was estimated.

### Real-time PCR

Total RNA was isolated using RNeasy Micro kit (Qiagen, Hilden, Germany) according to the manufacturer’s instructions. cDNA was synthesized using a PrimeScript RT reagent kit (Takara, Dalian, China) following the manufacturer’s protocol. The reference gene was *ACTB*. The primer sequences for *ACTB*, *LIG4*, *MRE11*, *DCLRE1C*, *XRCC4*, *XRCC5*, *XRCC6*, *XRCC7*, *NBN*, *RPA3*, *RAD50*, *RAD51*, *RAD52*, *SPRIDR*, *BRCA1*, and *BRCA2* are shown in Table 2. Each 10 μL of PCR reaction contained approximately 5 ng of cDNA, 0.25 μM of each forward and reverse primers, and 5 μL of SYBR Green I Master (Thermo Fisher Scientific). The enzyme was activated at 95 °C for 5 min, followed by 45 cycles of 95 °C for 10 s, 60 °C for 5 s, and 72 °C for 10 s. The tested genes were relatively quantified with an Eco^TM^ real-time PCR system (Illumina, San Diego, CA, USA) and normalized to the reference gene by the standard curve based method. The cDNAs were amplified twice and in triplicate on each plate.

### EGFP reporter-based assay

The eGFP reporter plasmids was digested with HindIII (Thermo Scientific) for 2 h and purified by ethanol precipitation. Aliquots were analyzed by gel electrophoresis to confirm complete digestion. The porcine fetal fibroblasts were electroporated with 4 μg of linearized eGFP reporter plasmid or 4 μg of linearized pEGFP-N1 as positive control. The transfected cells were plated and allowed to attach overnight. Cells were then treated with small molecules at indicated concentrations for 48 h. Expression of eGFP was monitored by Accuri C6 Flow Cytometer (BD Biosciences, San Jose, CA, USA). eGFP repair efficiency, which represents HDR efficiency, was calculated as the ratio of eGFP-positive cells over total cells.

### Evaluation of knock-in events

Porcine fetal fibroblasts were co-electroporated with *ROSA26* template donor and pX330-R5 plasmid. After cell adherence, transfected cells were treated with small molecules at the indicated concentrations for 48 h. Cells were incubated without small molecules for an additional 24 h, and then screened with 400 μg/ml G418 for approximately two weeks. All formed cell colonies were picked and cultured in 48-well plates. When confluency was reached, the cell colonies were subcultured, and 10% of each colony was lysed individually for genotyping in 10 μL of lysis buffer (0.45% NP-40 with 2 mg/mL Proteinase K) for 1 h at 56 °C and then 10 min at 95 °C. The lysate was directly used as template for PCR. PCR reaction was performed using the PrimeSTAR Max DNA polymerase (Takara, Dalian, China) under the following conditions: 98 °C for 5 min; 35 cycles of 98 °C for 10 s, 58 °C for 5 s, and 72 °C for 10 s/kb; 72 °C for 2 min; and held at 4 °C. PCR primers are shown in Table 2. PCR products were analyzed by gel electrophoresis on 1.5% agarose gel. The sequences of the individual colony were determined by Sanger sequencing.

### Statistical analysis

The data for gene expression, cells viability, cell cycle distribution and HDR efficiency were analyzed by one-way ANOVA with PASW Statistics 18 (IBM SPSS, Chicago, IL, USA). Data are expressed as means ± SEM. The level of significance was set at *P* < 0.05.
